# Forecast Model Update Based on a Real-Time Data Processing Lambda Architecture for Estimating Partial Discharges in Hydrogenerator

**DOI:** 10.3390/s20247242

**Published:** 2020-12-17

**Authors:** Fabio Henrique Pereira, Francisco Elânio Bezerra, Diego Oliva, G.F.M. de Souza, Ivan Eduardo Chabu, Josemir Coelho Santos, Shigueru Nagao Junior, Silvio Ikuyo Nabeta

**Affiliations:** 1 Informatics and Knowledge Management Graduate Program, Nove de Julho University—UNINOVE, São Paulo 01525-000, Brazil; oliva_diego@uni9.edu.br; 2 Industrial Engineering Graduate Program, Nove de Julho University—UNINOVE, São Paulo 01525-000, Brazil; elanio@uni9.edu.br; 3 Polytechnic School, University of São Paulo—EPUSP, São Paulo 05508-010, Brazil; gfmsouza@usp.br (G.F.M.d.S.); ichabu@pea.usp.br (I.E.C.); josemir@pea.usp.br (J.C.S.); snjunior@usp.br (S.N.J.); nabeta@pea.usp.br (S.I.N.)

**Keywords:** autoregressive forecasting model, lambda architecture, partial discharges, power hydrogenerators, real-time data processing

## Abstract

The prediction of partial discharges in hydrogenerators depends on data collected by sensors and prediction models based on artificial intelligence. However, forecasting models are trained with a set of historical data that is not automatically updated due to the high cost to collect sensors’ data and insufficient real-time data analysis. This article proposes a method to update the forecasting model, aiming to improve its accuracy. The method is based on a distributed data platform with the lambda architecture, which combines real-time and batch processing techniques. The results show that the proposed system enables real-time updates to be made to the forecasting model, allowing partial discharge forecasts to be improved with each update with increasing accuracy.

## 1. Introduction

There are many recent studies that address the development of forecasting models in the context of a monitoring system for industrial and electrical equipment, based on the analysis of temporal signals obtained through sensors [1,2,3,4,5]. In fact, real-time monitoring systems form one of the foundations of Industry 4.0 and have been developed to monitor various variables in industrial plants, electric generators/motors, and various other equipment [6]. These systems become more and more popular with the advances in Internet of Things (IoT) and Cloud technologies, especially in the areas of manufacturing and maintenance, but mainly aimed at visualizing the data collected due especially to the difficulties of real-time processing [6,7].

In general, forecast models are trained with historical data and, even with the acquisition of new data, they are not automatically updated to improve your forecasts. New data are used to assess the quality of the predictions made by the model and, if necessary, a new training process is started with the updated historical data set [1].

On the other hand, sensor data represent a significant opportunity for streaming and collecting from different devices every second, which creates important challenges on how to manipulate that data and perform data analysis online, given that current approaches are often limited by capacity, costs, and resources [8]. In this context, the data platform based on the lambda architecture stands out, combining traditional batch data processing with a fast, real-time data flow and it represents the state of the art in online data processing architectures [9].

This article presents a proposal to update a prediction model to improve forecast accuracy in the context of a distributed data platform based on the lambda architecture that combines real-time and batch processing techniques. The main contribution of the proposed system is the ability to combine the real-time and batch processing layers of the lambda architecture to update the forecasting model, with application in the estimation of partial discharges in power hydrogenerators. Partial discharges (PDs) are electrical discharges that occur inside or outside the insulation of a high-voltage system under electrical stress [10,11]. The proposed forecasting model is based on a long short-term memory (LSTM) recurrent neural network and explores the benefit of such models in allowing an update of weights as new data become available [12].

We organized the rest of the paper as follows. Section 2 discusses related work and research regarding online monitoring systems and prediction models. An overview of lambda architecture and the theory regarding forecasting models are presented in Section 3. The proposed approach to update the artificial neural network-based prediction model on a lambda architecture for estimating partial discharges in an hydrogenerator is presented in Section 4. Experimental results are presented in Section 5 and Section 6 concludes the work.

## 2. Related Work

Autoregressive model is a way to capture autocorrelation and seasonality in a sequence of observations ordered over time (time series) [13]. The time series analysis has basically three objectives: to identify the nature of the represented phenomenon looking for a behavior pattern, such as the trend pattern, the existence of seasonal variation, outliers, structural changes, etc.; use the variation in one series to explain the variation in another series; and, finally, predict the evolution of the time series’ variable from a mathematical model that describes the behavior of the observations [14].

The literature shows several studies that seek to improve the accuracy of autoregressive forecasting models, and many of these studies have used techniques that can be categorized into two groups: statistical approaches, such as regression analysis and time series analysis, and techniques based on artificial intelligence (AI), such as multilayer neural network, deep learning neural network, fuzzy logic, support vector machine (SVM), least squares support vector machine (LS-SVM), random forest, and deep learning, among others.

In [15] a model was proposed combining advantages of the neural network with random weights and a support vector machine, which can capture more dynamic characteristics of the multivariate data of the time series by means of weighting samples. The mutual information is used to determine time delays, between the value of the variable at time *t*, X(*t*), and the value of the same variable at time *t*-τ, X(*t*-τ) with different τ (time delay), where the first local minimum value of mutual information corresponds to the time delay for time series. 

Other prediction models based upon LS-SVM have been proposed by [16,17], just to name a few. These works address problems in different areas, for example, detecting mechanical failures in induction motors such as [18] and estimating the concentration of gases dissolved in oil in transformers as in [16], among others.

Regarding partial discharges (PD), the application of prediction models is also widespread. In this sense, analysis of electrical insulation failures reveal that this kind of failure is the root cause of more than 60% of the damage to high-voltage equipment [19]. Thus, PD recognition has been a topic of interest to distinguish different sources of PD failure within energy appliance insulation systems [11,19,20,21,22].

In [11] the authors proposed a new technique to classify PD’s patterns based on the learning of neural networks by ensemble (ENN). The ENN technique is based on training a series of neural network models with statistical parameters of partial discharge patterns and combining their predictions. By combining the outputs of the constituent neural networks through an aggregation unit using the dynamically weighted average strategy, a final assessment of partial discharge patterns is provided in relation to a range of partial discharge failure types. As another example of work on the topic we can cite [20], addressing a new method for locating PD in transformer windings.

Even in the context of partial discharges, the use of deep learning has received great attention and several contributions have shown that deep learning-based methods have better accuracy than typical machine learning methods, providing more efficient automated identification techniques. This kind of deep learning methods for PD classification was applied in [22,23], for example. In [22], a convolutional neural network (CNN) of multiple columns was used to incorporate ultra-high-frequency (UHF) spectra of multiple resolutions and a long-term memory network was used to merge information from built-in multisensors. Long short-term memory (LSTM) neural networks were also applied by [23] to classify partial discharges.

However, despite the evolution in the creation and precision of the forecasting model, the works generally do not describe mechanisms for manipulating and processing sensor data to update these models. This represents an important gap since improvements regarding the general applicability of the method can be made in a data acquisition process [21]. Additionally, the adoption of this type of mechanism for manipulating and processing sensor data is fundamental for the development of data-driven applications in the context of Industry 4.0. In this sense, some recent studies have presented proposals to adapt models to the experimental collected data, showing significant improvements in results. In [24], for example, the data-driven concept was used to adjust, optimize, and experimentally validate a model to optimize energy consumption on tower crane systems, presenting significantly superior results. Moreover, the implementation of efficient architectures for data collection, storage, and processing, possibly in a distributed way, can contribute significantly to mitigating the problems with limitations of computation abilities and communication channel bandwidth that are often described as complex tasks in data-driven practical control systems [25]. The severity of these limitations has motivated numerous researchers for the development and extensions of event-triggered control schemes such as those presented in [25,26,27,28], just to name a few.

According to [7], IoT and real-time, predictive maintenance systems face problems such as insufficient real-time data analysis, high cost to collect sensors’ data, and the configuration of fault detection rules. Such difficulties mean that the systems are often turned only to visualize the sensor data. The same authors suggest that the adoption of an architecture for real-time processing of sensor data, such as the lambda architecture, may favor the adoption of efficient preventive maintenance platforms.

In fact, lambda architecture is the state-of-the-art, real-time data processing technique [29]. However, many works on this topic are dedicated to the theoretical aspects of this architecture [9,30], with few recent real applications such as in E-Commerce Analytics [31] and intrusion and anomaly detection framework [32]. Authors in [7], for example, proposed a platform based on lambda architecture to accelerate automatic machine maintenance, but the application in a real situation was indicated as a future proposal of the work. Similarly, [6] proposed an industrial IoT system for monitoring electric motors in real time, in the Industry 4.0 context, and indicated the creation of a predictive model based on machine learning as a long-term future work.

The analysis of these studies shows the gap in contemporary literature and establishes the contribution of this paper. By comparing the previous literatures, the main contribution of the proposed system is the ability to combine the real-time and batch processing layers of the lambda architecture to update the forecasting model, with application in the estimation of partial discharges in power hydrogenerators. The proposed system makes it possible to collect, store, and process data separately in the batch and speed layers. The batch that handles large volumes of data layer is performed only sporadically while the speed layer is often executed with low data volume, ensuring model updates and mitigating common limitations of computation and communication channel bandwidth problems in data-driven, practical control systems [25,26,27,28].

From the point of view of the plant’s operation, the proposed methodology makes it possible to understand the evolution of the model’s forecasting capacity and to estimate the moment from which the forecast results of the model can be considered suitable for the application.

## 3. Theory

This section presents a concise description of the basic concepts used in this work and is necessary to understand the proposal.

### 3.1. Recurrent Neural Network

Hinton et al., in [33], published a paper on a neural network with deep learning, by which a fast learning algorithm was developed for deep belief networks for the first time. A recurrent neural network takes many forms, each highlighting a specific form of global feedback. However, they all incorporate a static multilayer perceptron or parts of it and exploit the nonlinear mapping ability of the multilayer perceptron [34]. 

**Figure 1:**
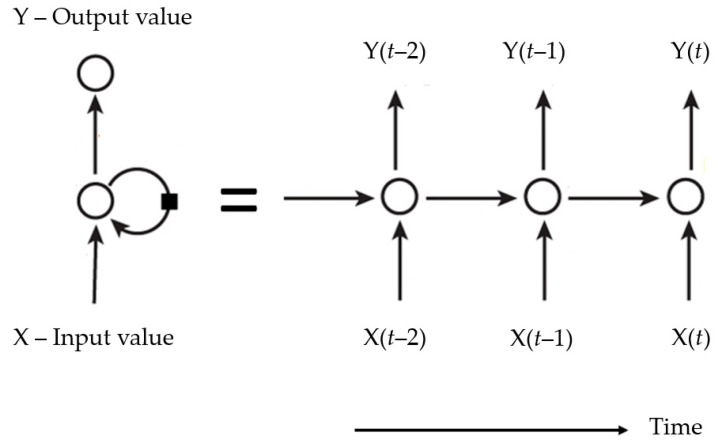
Simple structure of recurrent neural network.

Figure 1 shows a simple structure of Recurrent Neural Network (RNN), composed of a hidden layer, which receives inputs, producing an output and sending that output back to itself. If the RNN is expanded in a time frame, at each time step *t*, this recurring layer receives inputs X(*t*), as well as its own outputs from the previous time step Y(*t* − 2), Y(*t* − 1), and so on [21].

### 3.2. Lambda Architecture

The so-called Lambda architecture introduced by Nathan Marz in [35] is a state-of-the-art technology for real-time data processing. The architecture defines three layers, aiming at simplicity, scalability, fault tolerance, and robustness in data processing and information generation [9].

(1) The batch layer is designed to create an immutable master table with sensor data for the calculation of views with this data. This layer handles large volumes of data that are handled in batch and has high latency.
(2) The speed layer aims to compensate for the high batch layer latency by analyzing the data in real time. This layer processes the most recent data and updates the views created with the batch layer data set, solving the problem of data availability between consecutive batch layer calculations.
(3) Serving layer is responsible for merging the batch layer and speed layer information, to produce complete views. In general, the serving layer implies visions produced by the batch layer plus information produced by the speed layer in real time.

After each recalculation of the views in the batch layer, the redundant speed layer data are erased and the online processing of the most recent data resumes. Thus, the speed layer data are stored in temporary tables in small volumes and with low latency.

## 4. Proposed Approach

**Figure 2:**
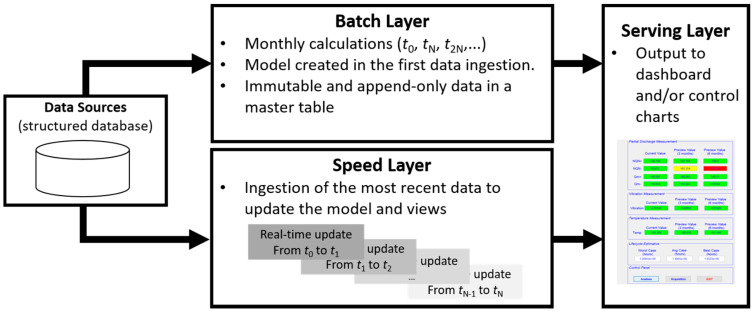
Structure of the proposed system with creation and update of the forecast model based on the lambda architecture. Weekly or monthly data ingestion in the batch layer and minute-wise or hour-wise in the speed layer.

In this section, the steps in the development of the proposed system are presented and described, from obtaining data of sensor readings to automatically updating the predictive model. The steps are described in the context of the lambda architecture and, therefore, consider the integration between the batch layer and the speed layer through the tools of the Hadoop ecosystem as well as the integration of this architecture with the prediction model. An illustration of the proposed system is shown in Figure 2.

### 4.1. Generation of Typical Partial Discharge Values

The partial discharge monitoring system that includes sensors, data architecture, and the autoregressive forecasting model is being implemented at the plant and, therefore, does not have enough data for a conclusive analysis of the hydrogenerator condition based on these variables and not even for the creation and evaluation of a forecasting model.

Thus, the methodology proposed here was evaluated based on a system simulation. Three scenarios were simulated for different hydrogenerator conditions in relation to the severity of partial discharges observed, defined according to [36]. In addition to evaluating the proposed methodology, the simulation performed is important to understand the evolution of the model’s forecasting capacity and to estimate the moment from which the forecast results of the model can be considered suitable for the application.

**Figure 3:**
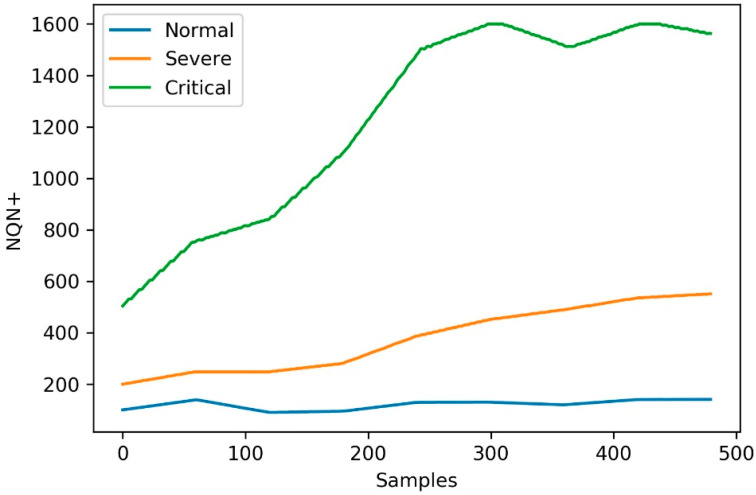
Data for different hydrogenerator conditions in relation to the severity of partial discharges.

The simulated scenarios considered the temporal evolution of the Normalized Quantity Number NQN+ in three operating conditions in relation to the severity of partial discharges, called *normal*, *severe*, and *critical* condition. The NQN+ values were obtained from the three conditions, as shown in Figure 3. The NQN+ values were extracted from the graphics in Figure 3 using a web-based plot digitizing tool for extracting data from a time series including XY coordinates, called WebPlotDigitizer [37]. In the WebPlotDigitizer tool, after loading the image and calibrating the axes, assuming that axes are perfectly aligned with image coordinates, the data were extracted automatically using the ‘X step with interpolation algorithm‘ available in the tool, with the parameters adjusted as described in Table 1 in order to get equivalent to a daily sample. The NQN values generated are available with this work as Supplementary Material.

The reading of the sensor data was done in 30 s, but the reading frequency usually occurs in a week-wise manner. However, for the start of the system’s operation, a daily frequency was established to speed up the generation of a database for future application of the proposed methodology. Thus, the three scenarios considered simulated daily measurements of NQN + values.

The extracted values were used to represent NQN+ over a 16-month monitoring and analysis horizon, resulting in 480 samples, of which 360 samples were used to simulate the operation of the model over a 12-month period.

The three scenarios are described as follows.

- Scenario 1—normal: coils in relatively good condition since there is no general increase over time in the NQN values, with these values in the range between 100 and 200 as shown in Figure 3 (blue line). In this scenario, ingestion of data in the batch layer occurs every six months, ingestion of data in the speed layer is carried out every two weeks, and the frequency of reading the data is daily. Therefore, the model was created from a sample of approximately 180 observations and received 10 consecutive updates with samples of 18 values each.
- Scenario 2—severe: significant thermal deterioration or thermal cycling, resulting in delamination of the insulation. There is a fixed increase in NQN over time, with values ranging from 200 to 600, as shown in Figure 3 (orange line). Here the batch layer is executed every six months with around 180 values and the speed layer every 10 days. In this case, model updates occurred more frequently due to the severity of partial discharges.
- Scenario 3—critical: high NQN values indicating critical thermal deterioration and resulting in delamination and severely deteriorated coils. NQN values start around 500 and can reach a maximum value close to 1500 at which they tend to stabilize, although the winding continues to deteriorate, as shown in Figure 3 (green line). For this scenario, the model was created with data from six months of measurements (180 samples) and received weekly updates due to the critical state of partial discharges.

### 4.2. Data Ingestion into Hadoop Distributed File System 

The first step in the development of the proposed system concerns the data ingestion in Hadoop’s HDFS system from the data source. This ingestion task was performed using the Apache Sqoop tool, which is designed to transfer data between structured datastores and the HDFS file system. The tool used in this data ingestion task can vary depending on how the data collected by the sensors are stored. In this stage, two different types of sqoop jobs were created, one job to collect data in batches on a monthly basis, which may vary depending on the application, and another job for online collection as new data were generated.

### 4.3. Batch Layer and Speed Layer Creation in Hive

The ingestion of batch data was used in the creation of the so-called batch layer, which consolidates all the data collected immutably in a master table, to ensure the historical reliability of the data, and was used for the creation and training of the forecast model. The ingestion of data in the batch layer was carried out incrementally, usually on a monthly or weekly basis, but the forecasting model was created and training only in the first ingestion.

In the speed layer, on the other hand, data ingestion occurred in real time, collecting the data that was generated since the last batch layer update. In this case, the periodicity can be minute-wise also depending on the application and defined by the user.

The data collected in real time in the speed layer were used to update the training of the forecast model, as well as to provide the user with views of the current situation of the monitored equipment, together with the data of the batch layer, and views of the future situation of the equipment, together with the values estimated by the autoregressive forecasting model. Whenever the batch layer was updated, the speed layer data were deleted and the last-value parameter that indicates the starting point to restart the data reading for the new speed layer was also updated.

The structures for creating the batch layer as well as for the speed layer were tables created in the Hive data warehouse, developed for Apache Hadoop. As Hive is automatically connected to HDFS, views were updated whenever new data ingestion occurred in the speed layer, which also automatically updated the forecasting model. The automatic update can be managed with a workflow scheduler tool like Oozie, for example.

### 4.4. Forecast Model Creation

The proposed forecasting model was based on a LSTM recurrent neural network and it explored the possibility of updating the model as new data were generated. The creation and update of the model were carried out in the context of the lambda architecture, as illustrated in Figure 2.

The batch layer can be updated weekly or monthly and, therefore, ingested a large volume of data. These data can be used to present views to the user and other purposes, but only the first intake was used to create the forecast model. The model was created in the Python environment using the machine learning scikit-learn keras and tensorflow libraries with Apache Spark’s in-memory primitives.

In this paper we used Keras framework for presenting the structure of the deep neural network prediction model because it is currently one of the most used libraries for this purpose. The Keras2DML framework can be used later to the parallel model training on Apache Spark.

### 4.5. Updating the Forecast Model

The forecast model was updated in the context of the lambda architecture after each speed layer new ingestion. The user can define exactly what frequency of update is desired, depending on the type of data collected by the sensors.

In this work, we dealt with variables related to partial discharges, in addition to generator vibration and temperature. These values can be correlated so that a condition (or event) can be evidenced that allows guiding operators on probable failures in progress and, thus, assisting them in decision making and strategic planning of stops and preventive maintenance.

### 4.6. Monitoring of Partial Discharges

**Figure 4:**
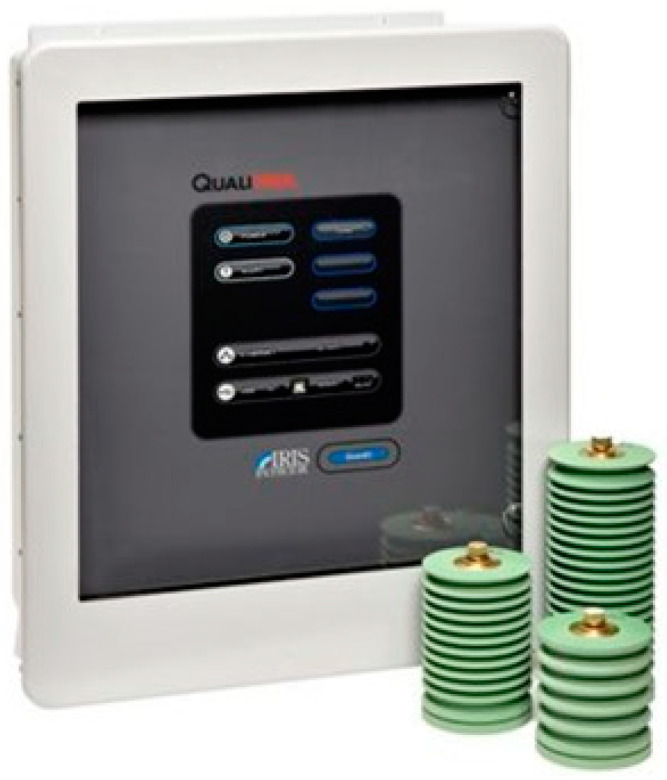
Power partial discharge epoxy mica capacitive sensors and GuardII monitoring system from Iris Power.

**Figure 5:**
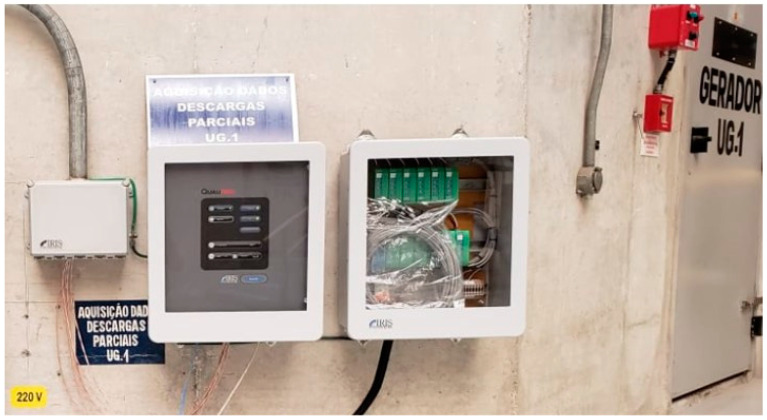
Installation panel for this system at the plant.

The partial discharge monitoring system was composed of power partial discharge epoxy mica capacitive sensors from Iris Power that were connected to the GuardII monitoring system for collecting of partial discharge pulse data through the sensors. Figure 4 and Figure 5 present, respectively, an illustration of this equipment and the installation panel for this system at the plant. Detailed information of the sensor including partial discharge pulse measurement, operating conditions, and testing and certification can be obtained in the Iris Power manual [38].

**Figure 6:**
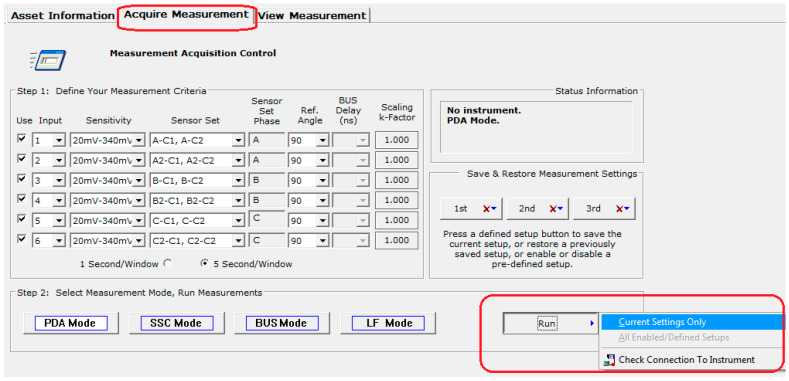
Holding register in the condition-based monitoring instrument GuardII for hydrogenerator.

The monitoring of partial discharges provides information about small electrical sparks that occur in the insulation due to its aging or deterioration. In this case, two important criteria were used to assess the operating conditions of the generator: The Normalized Quantity Number (NQN+ and NQN–) and the Maximum Amplitude Number (Qm+ and Qm–). The first data were obtained through the Holding Register on the condition-based monitoring instrument GuardII using the ModBus Transmission Control Protocol (TCP). The acquisition parameters were configured in the partial discharge sensors’ interface available in the system, according to Figure 6.

**Figure 7:**
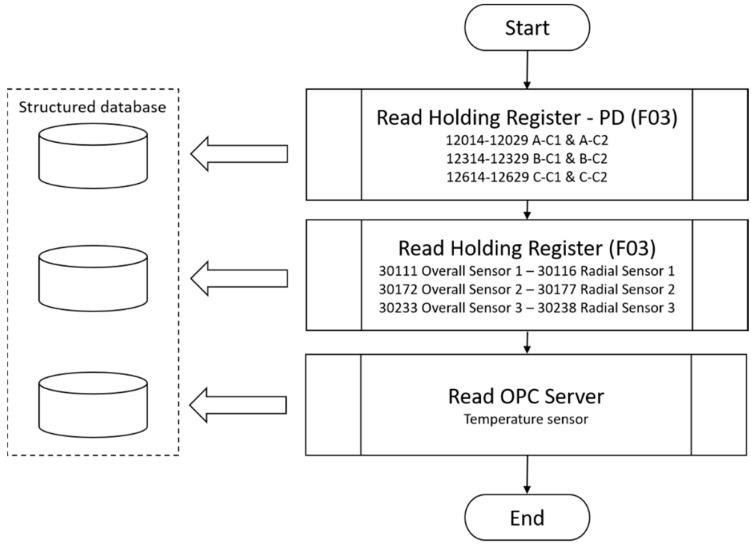
Diagram of sensor data acquisition. Three different sensor sets for partial discharges (A-C1 & A-C2, B-C1 & B-C2, C-C1 & C-C2), three sets of vibration sensors, and one set of temperature sensors.

After configuring the acquisition control parameters, the values were collected and stored in structured databases, as shown in the diagram in Figure 7, for partial discharges, temperature, and vibration. The temperature values were read from an open platform communications server, PCL Siemens S7, which specified the communication of real-time sensor data.

The stator end-winding vibration caused by the loosening of the windings, together with the thermal cycling that deteriorates the condition of the insulators, can cause the rupture of the copper conductors or even failures due to the wear of the insulation.

## 5. Experimental Results

This section presents the results of a simulation of an application of the proposed approach for updating a model for forecasting future values of partial discharges in the stator insulation system of an hydrogenerator.

**Figure 8:**
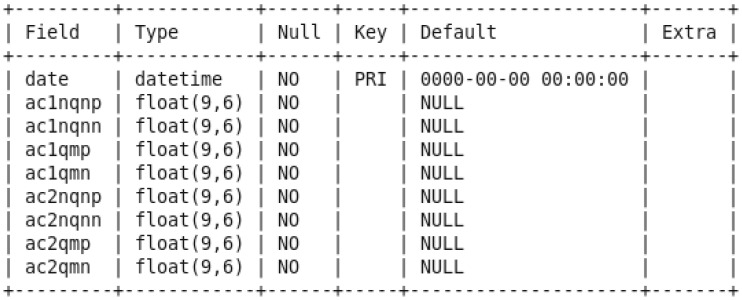
Structure of the table pddata in hydrogenerator_db database for values of partial discharge from the set of sensors A-C1 & A-C2 (variables NQN+, NQN–, Qm+, and Qm–).

First, we created two sqoop jobs to get the collected sensor data from a structured mysql database to the Hadoop HDFS. The partial discharge data were in a table called pddata from the hydrogenerator_db database, which has the structure illustrated in Figure 8, as an example for the set of sensors A-C1 & A-C2 and the variables NQN+, NQN–, Qm+, and Qm–.

**Figure 9:**
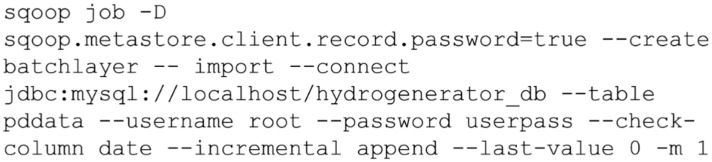
Script for creating the Sqoop batchlayer job to connect to the database and import data to the Hadoop Distributed File System (HDFS). Access password configured in the job itself for illustration purposes.

The next step of the process was to ingest data into Hadoop’s HDFS from hydrogenerator_db database to create the batch and the speed layers. We created two sqoop jobs to connect to the database and import data to the HDFS, according to the script in Figure 9 for batch layer, for example. 

Data ingestion was performed incrementally in HDFS according to the date parameter (primary key) and controlled by the last-value parameter that represented the last value read and, therefore, the starting point for the next import. In this simulation we considered a local database and the creation of a single process for the data ingestion task (*m* = 1). Sqoop imported the data to a directory in the default HDFS user directory created automatically with the same name as the source table.

Once the job was created it could be easily and repeatedly executed using the sqoop job –exec batchlayer command, with the job automatically saving the last-value parameter value for the next executions, ensuring that only the new data would be imported with each execution. It means the sqoop detected new registers in the database and updated the files in HDFS. In fact, the imported values were kept in HDFS in files in the comma-separated values (csv) format.

After data ingestion on HDFS, the next step was to create the master immutable table on Hive for the batch layer, batch views, and model training. Here we used the Beeline version 1.1.0-cdh5.13.0 as a wizard to run Hive queries [39]. The connection to Hive was accomplished using the following command:

!connect jdbc:hive2://

By default, Hive uses map reduce as a processing engine, which uses batch processing of large amounts of data. For our application, it was convenient to change the Hive processing engine to spark that works in memory and promotes a significant gain in relation to the processing time. The change of the execution engine can be made using the command 

set hive.execution.engine = spark

**Figure 10:**
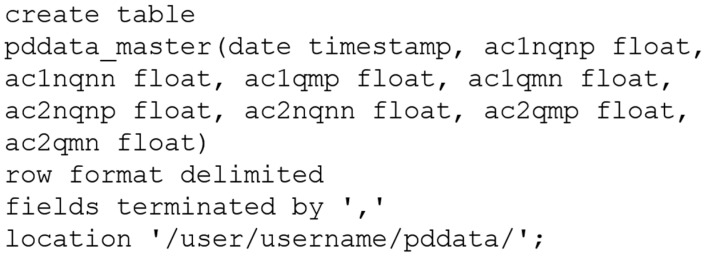
Script for creating master table in Hive, using a simple SQL-like query language. The location attribute must indicate the absolute path of the csv file in HDFS. The master table fields in Hive need not have the same names used in the file, but they must be created in the same original order.

Then, we created on Hive a database to store the structures for master table in batch layer. The database was created exactly with the same name as the table inserted into HDFS, pddata, and was linked to the file csv in HDFS. It means that the master table was updated whenever a new data ingestion occurred in HDFS by executing the sqoop job. The master table was created in Hive database using a simple SQL-like query language, as the script presented in Figure 10.

Again, it is important to note that the master table was automatically updated from any new importing data into HDFS. These data were used to create the forecast model, which can happen only once on first ingestion.

Model training can be performed using all or only part of the master table data set. It is possible, for example, to carry out the future forecast for all variables or for only one of the variables indicated in Figure 6. As it is batch processing, the data are transferred to the forecast model by means of a file. In this case, we used the beeline tool to save a hive query output to a file using command:

beeline -u ‘jdbc:hive2://’ –outputformat=csv2 –showHeader=false -e “hive.hql” > output_batchlayer_file.csv

in which “hive.hql” is a sequence of commands in the Hive SQL-like query language as, for an example, for variable A-C1 NQN+, “use pddata; select ac1nqnp from pddata_master;”.

In addition to training the forecasting model, data from the master table can be used to create views for the user as, for example, min and max values, first and third quartiles, and median values for box plot graphics.

A similar approach was applied to create the speed layer in Hive, with real-time processing. So, a sqoop job was created and executed to ingest the most recent data into Hadoop’s HDFS from hydrogenerator_db. In this case, however, two important points had to be considered: (1) As the Sqoop speedlayer job must acquire only the data generated since the last batch layer update, the last-value parameter must be updated whenever the batchlayer job is executed, at which point the speed layer data must be reset. This is done to ensure that there will be no duplicate data between the two layers. The value of the last-value parameter is defined after each execution of the batchlayer job using: var=$(mysql --user=root --password=userpass hydrogenerator _db -s -e “select max(date) from pddata;”) and the speed layer deleted by sqoop job --delete speedlayer. (2) When the sqoop speedlayer job ingests the data into HDFS a file with the same name as the batch layer is created. Therefore, to prevent the data collected online from overwriting the data in batch, a new directory must be created for speed layer, through the command: --target-dir.

**Figure 11:**
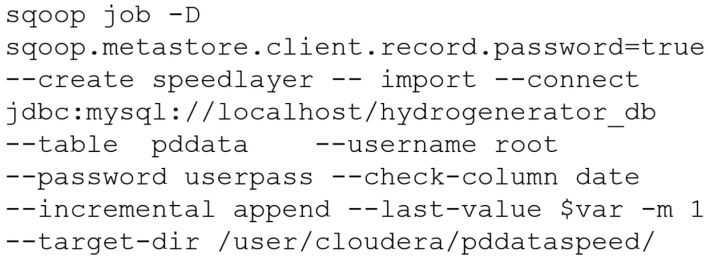
Script for creating the Sqoop speedlayer job to connect to the database and import data to the HDFS. Password configured in the job itself for illustration purposes.

The script for creating the Sqoop speedlayer job is presented in Figure 11. Unlike the batch layer, in the speed layer a temporary table was created in Hive, just for updating the forecast model and for creating views with data from the master table in the batch layer. The temporary table in the speed layer was created similarly to the master table, as shown in Figure 10.

Finally, we applied the speed layer data saved in the local file output_speedlayer_file.csv to update the model’s training and improve its forecasting capacity, as discussed below.

### Creating and Updating the Forecast Model

This section presents the results for creating and updating the forecast model using data from the batch and speed layers, respectively, as an example for variable A-C1 NQN+. The first phase was to divide the data into training and test samples and turn them into a supervised learning problem, in which previous time step samples were used as explanatory variables for the current time step sample. Also, in this phase we rescaled data to have values between –1 and 1.

Thereafter, in each scenario the model was updated as new data became available in the speed layer. The model’s precision results before and after the updates were then presented and compared. In this simulation, a new sample was generated once a day, the batch layer job ran every six months, collecting 180 NQN+ values in each execution, and the speed layer was executed according to the severity of partial discharges.

**Figure 12:**
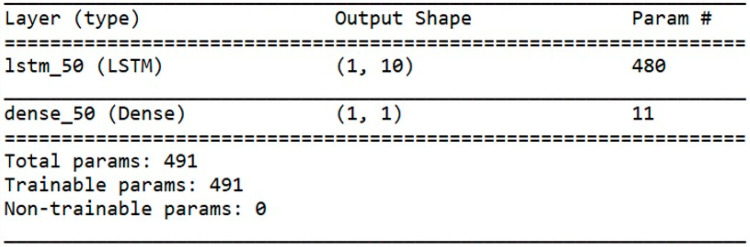
Summary of the proposed model.

The prediction model was based on a recurrent long short-term memory network (LSTM) with a backpropagation algorithm and the default sigmoid activation function. The model had an input layer with 1 neuron, a hidden layer with 10 LSTM blocks or neurons, and a single prediction output layer. The model was fit for 1000 epochs with online training (batch size of 1), Adaptive moment estimation (ADAM) optimization algorithm, and the root mean squared error (RMSE) loss function. A summary of the model is illustrated in Figure 12.

The values of the model parameters were defined experimentally and results of the precision of predictions as well as the computational time as a function of the number of neurons in the hidden layer are shown in Table 2, as an example for the third scenario. It is possible to see from the average values in the last row of the table that the use of 10 neurons in the hidden layer of the network produced the best results both in terms of RMSE and in relation to the computational cost in the training stage. This value was adopted in the following experiments. Other parameters did not produce significant changes in the results.

In each update the new data collected by the speed layer were added to the database, divided into training and test sets (80–20%), and the model evolved for an additional 50 epochs. The effects of the variation in the number of epochs for the model updating were also evaluated and are shown in Table 3 for scenario critical. Results in the table show that this parameter had a great influence on the computational cost of the model without, however, promoting a significant performance gain. All experiments were performed on a Dell Inspiron 14 series 5000, Intel Core i7, 16 GB of RAN (Random Access Memory) computer from Dell Technologies Inc., Round Rock, Texas, United States of America, using IDE Spyder (Python 3.7).

**Figure 13:**
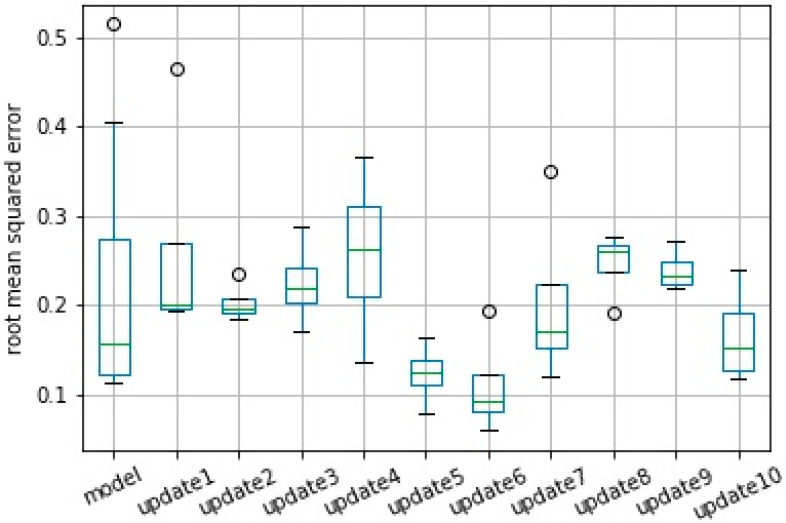
Performance based on the root mean squared error (RMSE) from original model and from consecutive updates. Results from 10 model runs for scenario normal.

**Figure 14:**
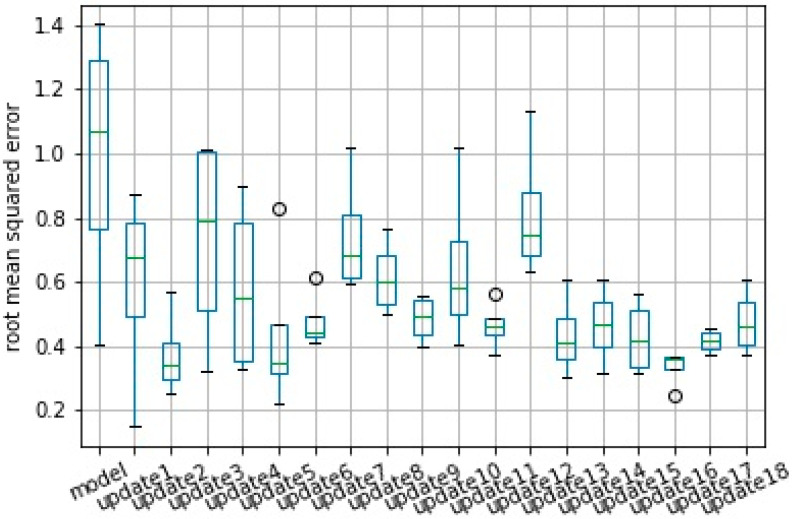
Performance based on RMSE from original model and from consecutive updates. Results from 10 model runs for scenario severe.

Performance of the prediction model evaluated by the RMSE is presented using a boxplot from 10 runs. Results from the original model, which was created with batch layer data, and from all consecutive model updates are presented in Figure 13 and Figure 14 for scenarios normal and severe, respectively.

It is important to note that the mean error obtained in model training for scenario normal, which was 0.222, represents a forecast accuracy of 99.7%, with a maximum error of 0.514 and an accuracy of 99.4%. The updates promoted a further small improvement in the model reaching 99.8% accuracy in relation to the RMSE in update 10. However, the reduction of the standard deviation of the error after the model updates stood out. The error of the original model had a deviation of 0.146, which was reduced to 0.055 after the 10th update, creating a more robust model.

For scenario 2—severe, the average error was reduced from 0.986 to 0.475, which represents an increase in model accuracy from 99.6% to 99.8%. This accuracy here was calculated as 1—nRMSE in which the nRMSE is the normalized RMSE defined according Equation (1).

nRMSE = RMSE/mean(Y*_t_*) (1)

in which mean(Y*_t_*) is the average of the target values in the test set.

Also, in this case, the reduction of the standard deviation of the error was the most significant, reducing from 0.444 in the original model to 0.104 in the last update.

**Figure 15:**
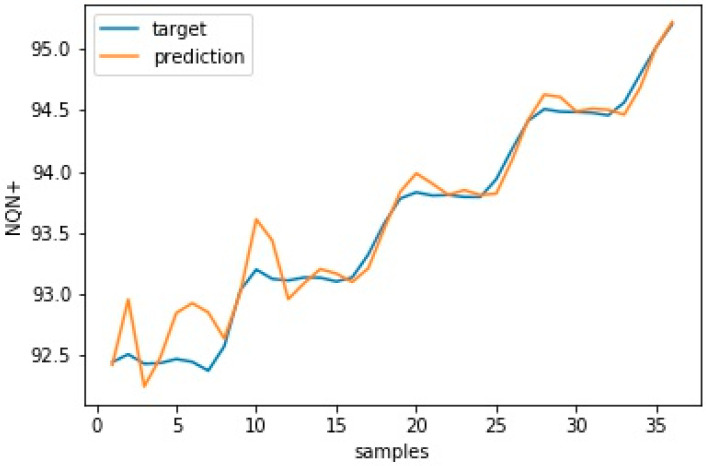
Target versus prediction for scenario 1—normal training data.

**Figure 16:**
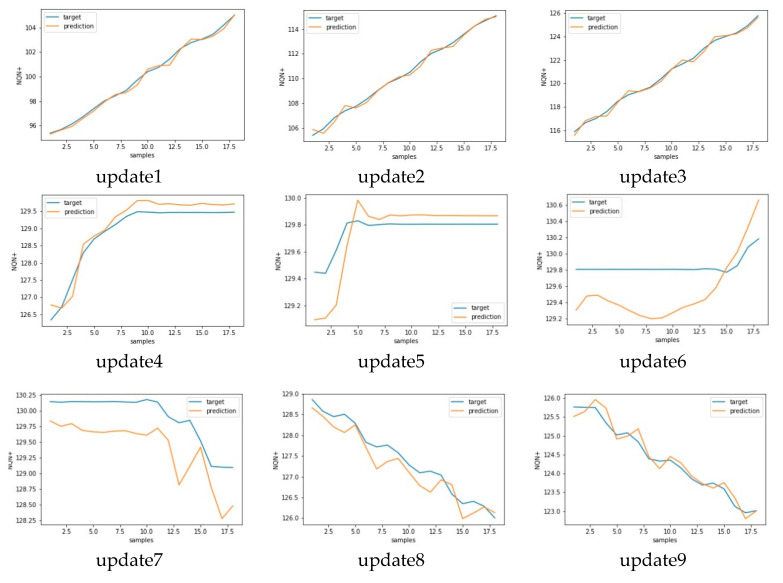
Target versus predicted values for nine consecutive updates for scenario 1—normal.

It is worth noting that the RMSE did not consider variations in the data collected in the speed layer and that this variation interfered with the accuracy of the model. Thus, even if an update improves the forecasting capacity of the model, the RMSE value may be higher than the previous error due to the variance of the new data used to evaluate this update. Figure 15 and Figure 16 show the model’s validation results with plots for target versus prediction data for training and test data for scenario 1—normal.

**Figure 17:**
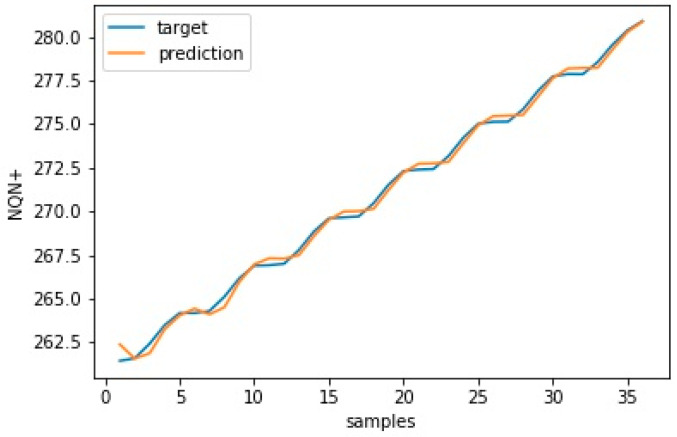
Target versus prediction for scenario 2—severe training data.

**Figure 18:**
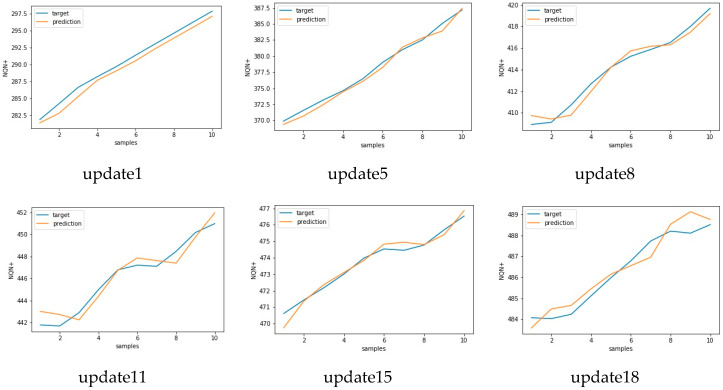
Target versus predicted values for six updates for scenario 2—severe.

**Figure 19:**
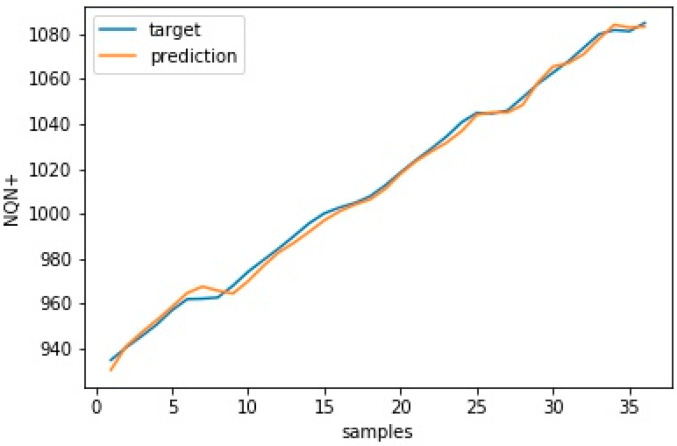
Target versus prediction for scenario 3—critical training data.

**Figure 20:**
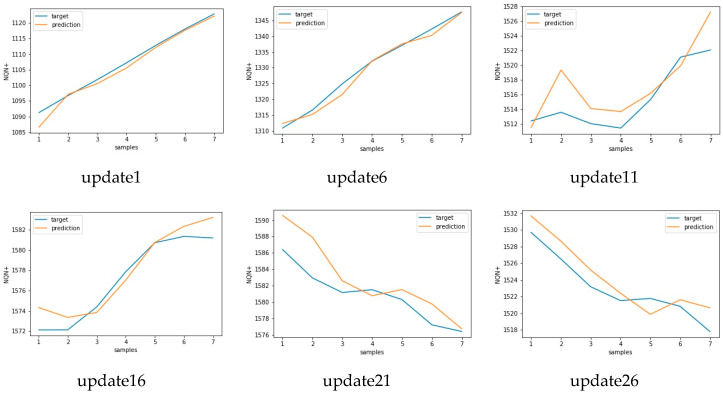
Target versus predicted values for six updates for scenario 3 - critical.

Similar results for scenario severe and critical are presented in Figure 17, Figure 18 and Figure 19, Figure 20, respectively. As in these two scenarios, the number of updates was greater than in scenario 1. Only a few updates are shown in the Figure 18 and Figure 20.

For scenario 3, the initial updates promoted an improvement in the RMSE obtained by the model, reducing the original model average error from 1708 to 1044 in update 17. This reduction in the average error represents a change in accuracy from 99.7% to 99.8%, which shows that there was little margin for improvements in the model. The standard deviation of RMSE was practically stable in this case.

**Figure 21:**
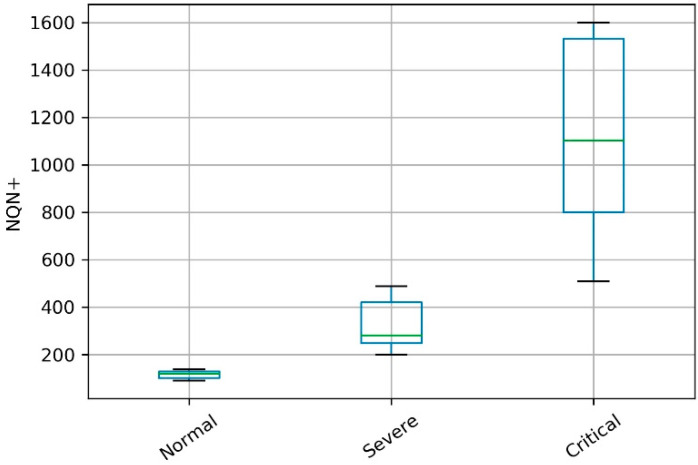
Illustration of the behavior of the datasets in the three scenarios.

It is important to note that the values for scenario 3 had a greater standard deviation in relation to the other two scenarios, as illustrated in Figure 21 as a boxplot format. But despite this greater variation, the accuracy of the model for this scenario was greater than 99% as well in the two other scenarios. This result indicates that the model presented low sensitivity in relation to variations in the severity of the partial discharge, presenting a competitive performance with the related literature regardless of the scenario tested: normal, severe, or critical conditions.

**Figure 22:**
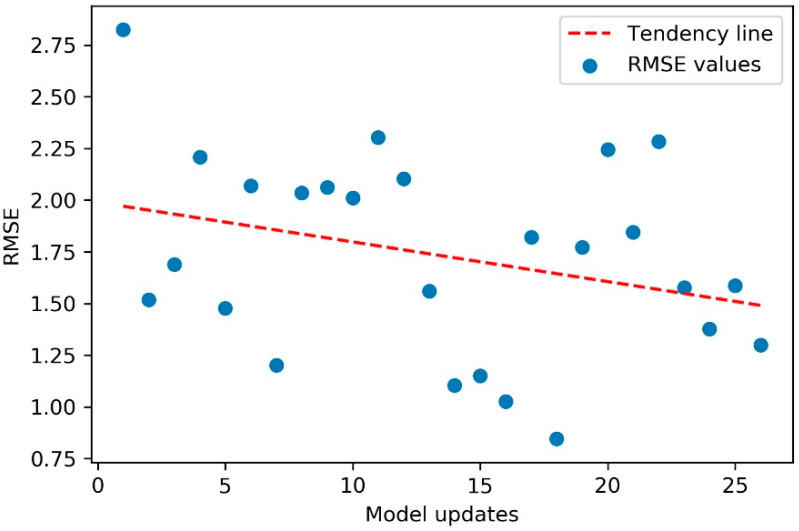
Scatter plot of RMSE values for scenario 3—critical. Tendency line shows that the model tended to stay more accurate as new updates were performed.

In addition, despite variations observed in the RMSE values, and a greater variation in the data for scenario 3, the model tended to increase in accuracy, as illustrated in the scatter plot of Figure 22.

Table 4 shows a comparison of the proposed approach with some works that used partial discharge, type of partial discharge, and recognition accuracy.

## 6. Conclusions and Future Work

This paper proposed a method to update the forecasting model aiming to improve its accuracy, based on a distributed data platform with the lambda architecture, which combines real-time and batch processing techniques. The architecture allows the creation of a prediction model for partial discharges in hydrogenerators using historical data from batch layer processing. In addition, real-time data in the speed layer were used to update the model, gradually increasing the accuracy of its predictions. After updates, the model evolved in its ability to estimate partial discharges, promoting an increase in the accuracy of the forecast. In addition, the robustness of the model’s prediction stood out, which showed low variation in all forecasts, with a low standard deviation in RMSE values.

The model presented low sensitivity in relation to variations in the severity of the partial discharge, presenting a competitive performance with the related literature regardless of the scenario tested: normal, severe, or critical conditions. The use of a batch processing layer and a real-time processing layer made it possible to handle large volumes of data (batch layer) while ensuring that the model was updated by frequent execution of the speed layer. This approach allowed us to mitigate common limitations of computation and communication channel bandwidth problems in data-driven practical control systems.

A disadvantage of the proposed approach is the use of RMSE since this absolute error measure has a high influence of outliers in data on the forecast performance evaluation. In addition, RMSE has low reliability as the results could be different in different fraction of data. The use of a relative measure can result in greater accuracy of the model, especially in the latest model updates in scenario 3.

Future work includes the application of a technique based on the wavelet transform to determine the optimal delay in the autoregressive forecasting model and the application of the proposed architecture for forecasting future values for the other partial discharge variables, as well as for vibration and temperature. The coupling of the model forecast results with the equations for calculating the remaining life can also be considered.

## Figures and Tables

**Table 1:** Parameters’ values used in WebPlotDigitizer tool.

Parameters	Normal	Severe	Critical
X_min	0	0	0
ΔX Step	16/480	16/480	16/480
X_max	16	16	16
Y_min	90	200	490
Y_max	150	600	1600
Smoothing	0	0	0

**Table 2:** Precision and computational cost of the model as a function of the number of neurons.

5 Neurons		10 Neurons		15 Neurons	
RMSE	Time (s)	RMSE	Time (s)	RMSE	Time (s)
0.163	180.51	0.159	165.54	0.146	206.19
0.173	180.14	0.144	183.05	0.151	211.07
0.167	196.40	0.127	189.83	0.186	215.06
0.312	230.22	0.260	182.78	0.139	235.90
0.167	252.68	0.311	176.38	0.175	259.87
0.149	193.86	0.111	180.52	0.140	181.97
0.804	190.24	0.292	182.36	0.147	190.76
0.371	238.93	0.150	183.14	0.152	192.31
0.137	207.85	0.137	175.30	0.157	203.90
**0.271**	**207.87**	**0.188**	**179.88**	**0.155**	**210.78**

**Table 3:** Computational performance of the model as a function of the number of epochs in the update steps: example for scenario 3—critical. The last line illustrates the average values.

Epochs = 50		Epochs = 100		Epochs = 150	
RMSE	Time (s)	RMSE	Time (s)	RMSE	Time (s)
2.825	19.98	1.261	41.07	1.727	75.56
1.518	21.60	2.304	42.71	3.854	67.02
1.690	26.99	1.788	43.32	2.039	72.63
2.208	23.65	2.224	55.91	1.588	75.95
1.477	28.95	2.566	51.55	1.662	80.29
2.070	30.19	1.762	50.96	1.707	90.69
1.202	26.28	1.667	50.73	1.193	102.08
2.036	26.29	1.696	54.86	2.133	102.57
2.061	27.17	1.295	55.05	2.619	105.59
2.012	27.97	2.582	59.75	3.698	115.04
2.304	30.36	3.937	62.70	2.983	112.46
2.103	31.63	3.284	61.56	1.223	106.85
1.559	32.91	3.857	63.28	1.779	108.95
1.104	31.09	2.659	65.16	1.124	111.84
1.150	31.99	1.381	68.30	1.619	122.18
1.025	32.53	1.647	64.81	1.065	137.84
1.822	33.47	3.721	68.70	1.632	113.87
0.845	34.42	1.416	72.69	1.881	130.62
1.773	34.74	2.229	74.75	1.905	125.92
2.244	36.93	5.092	83.97	1.815	141.41
1.846	36.47	2.185	82.38	1.914	158.58
2.284	37.39	2.418	84.95	2.313	152.34
1.577	38.29	2.849	87.68	1.767	146.24
1.376	38.56	2.294	91.50	2.495	156.98
1.588	40.16	2.411	95.36	1.553	160.46
1.298	40.40	3.861	82.237215	2.207	155.15
**1.731**	**31.55**	**2.476**	**66.00**	**1.98**	**116.51**

**Table 4:** Comparison of approaches for partial discharge recognition.

Authors	Approach	Type of Partial Discharge	Recognition Accuracy
Barrios et al. 2019 [21]	Deep Learning Methods	Protrusion defect, particle defect, contamination defect and gap defect	95.6%
Li et al. 2018 [22]	Multi-Resolution Convolutional Neural Network	Artificial PDs in GIS tank	98.2%
Adam et al. 2018 [23]	Multiple PD Sources by Signal Features and LSTM	Corona, Surface, Needle-Plane and Void	97.04% for the test and97.38% on the training
Khan et al. 2016 [40]	PCA and artificial neural network	Ten defects related to partial discharge.	88%
Nguyen et al. 2018 [41]	Recurrent Neural Network	Overall, Corona, Floating, Particle, Void, Noise	96.74%, 97.04%, 79.54%,93.18%, 99.94%, 98.26%
Darabad et al. 2015 [42]	Data mining method for power transformer defect models using SOM and PCA	Ten defects related to partial discharge.	Grouped data visualization
Karimi et al. 2019 [43]	Deep Belief Networks	Corona, Surface, 1 Void, 2 Voids, 3 Voids, 4 Voids	96.6% to 99.8%
Yang et al. 2020 [44]	Spherical CNN, DCNN, SVM and BPNN	Protrusion, Particle, and Void discharge, Surface discharge, Overall	92.25% Spherical CNN, 62.25% DCNN, 90.25% SVM, 85.5% BPNN
Peng et al. 2019 [45]	A Convolutional Neural Network-Based Deep Learning Methodology	Five different types of defects	92.57%
This approach	Recurrent Neural Network and lambda architecture	PD in transformer windings	99.4% to 99.8%

## Supplementary Materials

Supplementary files available at https://www.mdpi.com/1424-8220/20/24/7242/s1. DatasetCritical excel file; DatasetNormal excel file and DatasetSevere excel file.
